# A meta-analytic evaluation of cholesteryl ester transfer protein (*CETP*) C-629A polymorphism in association with coronary heart disease risk and lipid changes

**DOI:** 10.18632/oncotarget.12898

**Published:** 2016-10-25

**Authors:** Shouwei Lin, Ruozhu Dai, Rong Lin

**Affiliations:** ^1^ Department of Cardiology, Fujian Medical University Affiliated First Quanzhou Hospital, Fujian Province, P.R. China

**Keywords:** coronary heart disease, cholesteryl ester transfer protein, polymorphism, association, meta-analysis

## Abstract

Lipid metabolism plays an essential role in the pathogenesis of atherosclerosis, a major cause for coronary heart disease (CHD). Cholesteryl ester transfer protein (CETP) is an important glycoprotein involved in lipid metabolism by transferring cholesteryl esters to apolipoprotein B-containing lipoproteins in exchange for triglycerides. The objective of this meta-analysis was to evaluate the association of *CETP* C-629A polymorphism with CHD risk and lipid changes. Four public databases were searched, and data from 17 qualified articles were extracted in duplicate and analyzed by STATA software. Overall association of C-629A with CHD risk was nonsignificant in 5441 patients and 7967 controls. Subgroup analyses by ethnicity revealed significance only in Caucasians, with the odds of CHD being 1.18, 1.43 and 1.41 under allelic, genotypic and dominant models, respectively (*P* < 0.001). Similarly, the -629C allele increased the corresponding risk of myocardial infarction by 1.23-, 1.28- and 1.29-fold (*P* < 0.02). The association of C-629A with CHD was significantly strengthened in prospective and large studies. Moreover, carriers of the -629C allele had significant higher levels of circulating CETP (weighted mean difference [WMD]: 0.45 μg/mL; 95% confidence interval [CI]: 0.25 to 0.65; *P* < 0.001), but lower levels of high-density lipoprotein cholesterol (HDL-C) (WMD: -3.65 mg/dL; 95% CI: -5.59 to -1.70; *P* < 0.001) relative to the -629AA homozygotes. The probability of publication bias was low. Our meta-analytic findings collectively demonstrate that the -629C allele was significantly associated with an increased risk of CHD in Caucasians, and this association may be mediated by its phenotypic regulation on circulating CETP and HDL-C.

## INTRODUCTION

It is widely accepted that lipid metabolism plays an essential role in the pathogenesis of atherosclerosis, a major cause for coronary heart disease (CHD) [1, 2]. The past decade has witnessed substantial advances in understanding the genetic basis of lipid abnormalities of biomedical importance. In particular, cholesteryl ester transfer protein (CETP) is a plasma glycoprotein involved in lipid metabolism, and it can trigger the transfer of cholesteryl esters from high-density lipoprotein (HDL) to apolipoprotein B-containing lipoproteins in exchange for triglycerides, a key step known as ‘reverse cholesterol transport’ [3]. The gene encoding CETP is shipped with 2056 polymorphic loci ( https://www.ncbi.nlm.nih.gov/gene/1071), and some of them have been proposed as potential regulators of CETP deficiency and HDL cholesterol (HDL-C) increase, as indicated by a comprehensive meta-analysis of 92 published studies [4]. However, the exact mechanism whereby *CETP* genetic loci alter susceptibility to CHD remains largely unknown. A clear understanding of how *CETP* genetic loci regulate lipid metabolism associated with CHD is therefore a challengeable task. To take a step further, we in this study meta-analytically evaluated the association of a promoter functional polymorphism, C-629A (rs1800775) in *CETP* with CHD risk and lipid changes. This polymorphism was reported to be a Sp1/Sp3 transcription factor binding site that can regulate the transcriptional activity of human *CETP* promoter [5, 6].

## RESULTS

### Eligible articles

**Figure 1:**
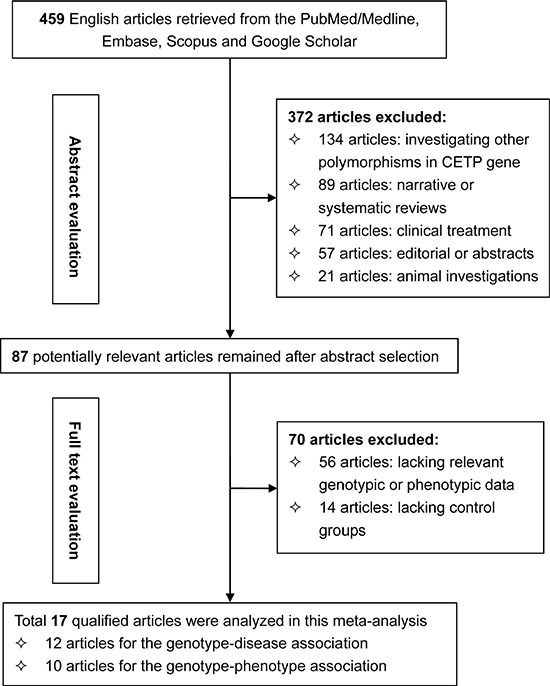
Flow diagram depicting the steps of article selection for this meta-analysis

Figure 1 is a flow diagram that depicted the steps of filtrating articles for this meta-analysis. From 459 initially identified articles from 4 electronic databases, 17 that satisfied our eligibility criteria were finally analyzed [7–24]. There were 12 qualified articles including 16 study groups (5441 CHD patients and 7967 controls) for the association between *CETP* C-629A polymorphism and CHD risk [7–18]. There were 10 qualified articles including 20 study groups (22488 subjects) for the relationship between *CETP* C-629A polymorphism and circulating lipid changes [13, 15–17, 19–24].

### Study characteristics

Table 1 (A, B, C) summarizes the characteristics of all study groups, and Supplementary Table S1 provides the mean values of lipid concentrations under study across C-629A genotypes. For the genotype-disease association, 7 of 16 study groups were based on East Asians, 3 on Caucasians, 2 on Middle Easterns and 4 on mixed populations. Coronary stenosis was assessed in 9 study groups and myocardial infarction in 7 study groups. Ten study groups enrolled controls from general populations and 6 from hospitals. Twelve studies were in retrospective designs and 4 in prospective designs. Age was reported to be matched between CHD patients and controls by 11 studies (Table 1).

**Table 1A:** The baseline characteristics of all eligible articles for the genotype-disease association

Author (year)	Ethnicity	CHD subtype	Source	Design	Matched	Genotyping	Patients	Controls
Eiriksdottir G (2001)	Caucasian	Myocardial infarction	Population	Prospective	NR	RFLP	388	794
Freeman DJ (2003)	Caucasian	Myocardial infarction	Population	Prospective	YES	Non-RFLP	498	1108
Tobin MD (2004)	Caucasian	Myocardial infarction	Hospital	Retrospective	NA	Non-RFLP	547	505
Zheng K (2005)	Asian	Coronary stenosis	Hospital	Retrospective	YES	Non-RFLP	203	209
Zee RY (UK) (2006)	Mixed	Myocardial infarction	Population	Prospective	YES	Non-RFLP	547	505
Zee RY (PHS) (2006)	Mixed	Myocardial infarction	Population	Prospective	YES	Non-RFLP	523	2092
Meiner V (males) (2008)	Mixed	Myocardial infarction	Population	Retrospective	YES	Non-RFLP	321	308
Meiner V (females) (2008)	Mixed	Myocardial infarction	Population	Retrospective	YES	Non-RFLP	256	351
Tanrikulu S (2009)	Middle Eastern	Coronary stenosis	Hospital	Retrospective	YES	RFLP	120	120
Poduri A (2009)	Asian	Coronary stenosis	Population	Retrospective	YES	RFLP	265	150
Padmaja N (2009)	Asian	Coronary stenosis	Hospital	Retrospective	YES	RFLP	504	338
Ghatreh Samani K et al (2009)	Middle Eastern	Coronary stenosis	Hospital	Retrospective	YES	RFLP	187	136
Wang J (2013)	Asian	Coronary stenosis	Hospital	Retrospective	YES	Non-RFLP	420	424
Lu Y (Chinese) (2013)	Asian	Coronary stenosis	Population	Retrospective	NR	RFLP	442	383
Lu Y (Malays) (2013)	Asian	Coronary stenosis	Population	Retrospective	NR	RFLP	110	155
Lu Y (Indian) (2013)	Asian	Coronary stenosis	Population	Retrospective	NR	RFLP	110	389

Notes: CHD, coronary heart disease; NR, not reported; RFLP, restriction fragment length polymorphism; BMI, body mass index; TG, triglycerides; TC, total cholesterol; HDL-C and LDL-C, high- and low-density lipoprotein cholesterol; CETP, cholesteryl ester transfer protein; Apo-AI, apolipoprotein AI; Apo-B, apolipoprotein B.

**Table 1B:** The demographic characteristics of all study populations for the genotype-disease association

Age (years)		Gender		BMI (kg/m2)		Smoking		Dyslipidemia		Hypertension		Diabetes	
Patients	Controls	Patients	Controls	Patients	Controls	Patients	Controls	Patients	Controls	Patients	Controls	Patients	Controls
71.0	76.0	1.000	1.000	27.30	26.00	NR	NR	NR	NR	NR	NR	NR	NR
56.9	56.7	1.000	1.000	26.00	25.60	0.530	0.550	NR	NR	NR	NR	NR	NR
61.9	58.6	0.680	0.620	25.90	25.70	0.402	0.170	NR	NR	0.310	0.168	0.087	0.020
55.4	54.8	0.675	0.675	25.92	23.89	0.680	0.411	NR	NR	0.552	0.167	NR	NR
58.3	58.4	1.000	1.000	25.50	25.00	0.571	0.566	0.132	0.083	NR	NR	0.056	0.027
58.3	58.4	1.000	1.000	25.50	25.00	0.571	0.566	0.132	0.083	NR	NR	0.056	0.027
44.0	42.2	1.000	1.000	29.00	26.60	0.468	0.203	0.449	0.250	0.375	0.172	0.108	0.020
50.5	49.5	0.000	0.000	29.70	26.90	0.537	0.128	0.420	0.282	0.450	0.239	0.220	0.048
54.0	52.0	0.780	0.483	32.00	25.00	0.558	0.225	NR	NR	0.467	0.108	NR	NR
47.5	47.0	0.838	0.760	28.18	23.53	0.351	0.200	NR	NR	NR	NR	NR	NR
50.7	49.7	0.909	0.888	24.08	23.61	0.423	0.257	NR	NR	0.381	0.305	0.337	NR
54.6	52.8	NR	NR	27.10	26.90	NR	NR	NR	NR	NR	NR	NR	NR
66.0	66.0	0.398	0.396	24.30	24.20	0.510	0.323	NR	NR	0.488	0.387	0.210	0.120
59.3	42.7	0.781	0.539	24.24	23.00	0.529	0.174	0.326	0.463	0.691	0.082	0.433	0.027
59.1	40.7	0.761	0.913	26.14	25.17	0.490	0.527	0.275	0.564	0.727	0.041	0.626	0.034
60.4	42.4	0.835	0.622	24.90	24.77	0.438	0.136	0.211	0.643	0.618	0.098	0.618	0.064

**Table 1C:** The circulating lipid profiles of all study populations for the genotype-disease association

TG (mg/dL)		TC (mg/dL)		HDL-C (mg/dL)		LDL-C (mg/dL)		CETP (μg/mL)		Apo-AI (mg/dL)		Apo-B (mg/dL)	
Patients	Controls	Patients	Controls	Patients	Controls	Patients	Controls	Patients	Controls	Patients	Controls	Patients	Controls
101.86	94.77	232.02	228.15	44.08	43.70	NR	NR	NR	NR	NR	NR	NR	NR
173.61	162.98	273.78	271.46	41.38	44.08	194.12	191.42	NR	NR	NR	NR	NR	NR
NR	NR	NR	NR	NR	NR	NR	NR	NR	NR	NR	NR	NR	NR
142.60	120.46	193.74	189.48	45.24	50.66	95.13	84.69	NR	NR	119.00	125.00	111.00	107.00
NR	NR	NR	NR	NR	NR	NR	NR	NR	NR	NR	NR	NR	NR
NR	NR	NR	NR	NR	NR	NR	NR	NR	NR	NR	NR	NR	NR
247.40	197.50	NR	NR	38.20	43.30	NR	NR	NR	NR	NR	NR	NR	NR
217.90	160.50	NR	NR	47.40	59.40	NR	NR	NR	NR	NR	NR	NR	NR
164.00	136.00	201.00	204.00	38.00	47.00	131.00	129.00	NR	NR	NR	NR	NR	NR
189.25	138.81	203.55	147.22	35.51	41.15	130.18	78.31	NR	NR	NR	NR	NR	NR
147.10	120.30	195.70	169.04	40.90	40.62	122.90	115.92	NR	NR	NR	NR	NR	NR
190.90	184.90	176.60	172.60	36.70	38.90	101.60	96.70	1.98	2.31	120.50	125.90	104.30	102.70
NR	NR	182.52	158.16	46.40	47.56	109.44	94.74	NR	NR	NR	NR	NR	NR
121.35	162.98	176.72	224.67	37.51	54.14	107.50	138.05	NR	NR	119.47	144.40	89.78	106.20
133.75	185.12	177.88	226.22	35.19	46.02	111.76	143.08	NR	NR	115.29	129.29	103.64	121.36
114.26	170.95	164.35	216.94	34.03	42.92	99.77	139.99	NR	NR	110.01	135.85	102.30	122.87

CHD patients were slightly older than controls (mean age: 56.74 vs. 52.99 years, *P* = 0.053) and gender composition was comparable (*P* = 0.111). Mean levels of BMI (*P* = 0.005), smoking status (*P* < 0.001), hypertension (*P* = 0.001) and diabetes (*P* = 0.182) were significantly higher in CHD patients than in controls. By contrast, controls had significant higher levels of circulating HDL-C (*P* = 0.001) and Apo-AI (*P* = 0.026) than patients.

For the genotype-phenotype relationship, circulating HDL-C was investigated in 16 study groups and triglycerides in 9 groups, LDL-C in 9 groups, CETP in 4 groups, Apo-AI and Apo-B respectively in 3 groups, as shown in Supplementary Table S1.

### CETP C-629A polymorphism and CHD risk

**Figure 2:**
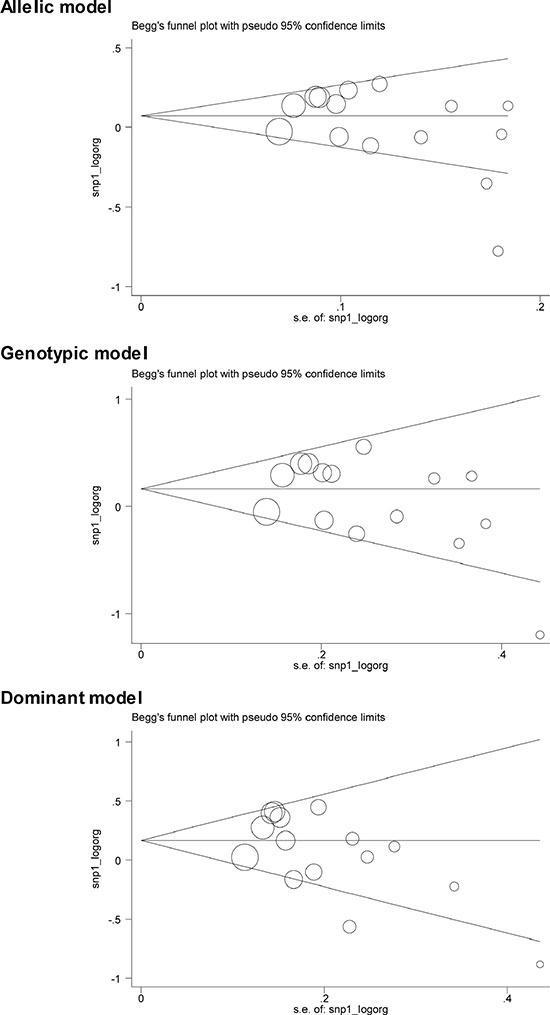
The Begg's funnel plots for the association of CETP C-629A polymorphism with CHD risk under three genetic models Each hollow circle in Begg's funnel plots denotes each study, and the size of circle is positively proportional to the sample size of each study.

Table 2 shows the overall and subgroup analyses of *CETP* C-629A polymorphism in association with CHD risk. In overall analyses, the -629C allele was nonsignificantly associated with a 4% (95% CI: 0.95 to 1.15; *P* = 0.412), 15% (95% CI: 0.98 to 1.35; *P* = 0.090) and 14% (95% CI: 0.99 to 1.31; *P* = 0.081) increased risk under allelic (-629C allele versus -629A allele), homozygous genotypic (-629CC genotype versus -629AA genotype) and dominant (-629CC genotype plus -629AC genotype versus -629AA genotype) models, respectively. These associations were obsessed by moderate heterogeneity, with the corresponding *I*^2^ statistic of being 69.1%, 50.2% and 60.4%. There was a low probability of publication bias except for dominant model (Egger's test: *P* = 0.055) (Figure 2). Additionally, in 5 studies involving only males, effect estimates were slightly reinforced relative to the overall estimates, and significance was detected under dominant model (OR = 1.22; 95% CI: 1.02 to 1.47; *P* = 0.033) with moderate heterogeneity (*I*^2^ = 53.0%).

**Table 2:** Overall and subgroup analyses of *CETP* gene C-629A in susceptibility to CHD under three genetic models

Groups	Studies	Allelic model				Homozygous genotypic model				Dominant model			
		OR	95% CI	P	I2 (%)	OR	95% CI	P	I2 (%)	OR	95% CI	P	I2 (%)
Overall	16	1.04	0.95 to 1.15	0.412	69.1	1.15	0.98 to 1.35	0.090	50.2	1.14	0.99 to 1.31	0.081	60.4
Males only	5	1.09	0.97 to 1.21	0.153	55.2	1.19	0.94 to 1.50	0.156	57.8	1.22	1.02 to 1.47	0.033	53.0
Ethnicity													
Asian	7	0.96	0.78 to 1.18	0.707	78.1	1.00	0.74 to 1.34	0.976	53.5	1.04	0.82 to 1.32	0.753	54.4
Caucasian	3	1.18	1.08 to 1.30	< 0.001	0.0	1.43	1.18 to 1.74	< 0.001	0.0	1.41	1.20 to 1.66	< 0.001	0.0
Mixed	4	1.08	0.92 to 1.27	0.363	67.8	1.17	0.83 to 1.64	0.374	68.6	1.21	0.93 to 1.56	0.153	64.8
Middle Eastern	2	0.89	0.55 to 1.44	0.644	73.2	0.96	0.52 to 1.78	0.901	34.2	0.79	0.40 to 1.52	0.474	72.1
CHD subtypes													
Coronary stenosis	9	0.95	0.79 to 1.14	0.562	75.2	1.00	0.77 to 1.28	0.976	45.0	0.97	0.76 to 1.22	0.776	61.7
Myocardial infarction	7	1.23	1.02 to 1.24	0.015	50.9	1.28	1.05 to 1.56	0.015	52.9	1.29	1.11 to 1.51	0.001	47.8
Source of controls													
Hospital	6	1.09	0.94 to 1.26	0.267	55.1	1.27	1.04 to 1.54	0.017	3.5	1.13	0.87 to 1.48	0.363	68.5
Population	10	1.02	0.89 to 1.16	0.790	75.1	1.10	0.88 to 1.37	0.413	62.6	1.13	0.95 to 1.35	0.166	58.5
Study design													
Retrospective	12	1.00	0.87 to 1.15	0.970	73.6	1.07	0.86 to 1.33	0.540	52.8	1.05	0.87 to 1.28	0.599	63.9
Prospective	4	1.21	1.01 to 1.25	0.038	46.9	1.27	1.02 to 1.59	0.037	49.7	1.28	1.07 to 1.54	0.007	47.3
Matched status													
YES	11	1.01	0.88 to 1.16	0.880	76.4	1.11	0.90 to 1.37	0.331	57.8	1.11	0.93 to 1.33	0.241	64.8
NR	5	1.11	0.99 to 1.24	0.072	25.0	1.23	0.96 to 1.57	0.104	32.3	1.17	0.91 to 1.52	0.220	57.8
Sample size													
< 500 subjects	6	0.85	0.65 to 1.12	0.251	7.9	0.85	0.58 to 1.24	0.407	44.2	0.84	0.62 to 1.15	0.285	48.2
≥ 500 subjects	10	1.12	1.04 to 1.21	0.004	46.2	1.25	1.07 to 1.46	0.005	44.0	1.25	1.09 to 1.43	0.001	51.8

Notes: OR, odds ratio; 95% CI, 95% confidence interval; NR, not reported.

Stratifying study groups by ethnicity identified significance only in Caucasians, with the odds of CHD being 1.18, 1.43 and 1.41 respectively under allelic, homozygous genotypic and dominant models (*P* < 0.001 for all), without observable heterogeneity (*I*^2^ = 0% for all). In contrast, the effect estimates were in an opposite direction, albeit nonsignificant in Middle Easterns across three genetic models.

In subgroup analyses by CHD subtypes, the -629C allele was observed to significantly increase risk of myocardial infarction by 1.23-, 1.28- and 1.29-fold respectively under allelic (*P* = 0.015), homozygous genotypic (*P* = 0.015) and dominant (*P* = 0.001) models, with borderline heterogeneity. By source of controls, the effect estimates were roughly comparable between hospital- and population-based studies, with significant heterogeneity.

By study design, the -629C allele seemed to confer a 21% to 28% increased risk for CHD in prospective studies across three genetic models (*P* < 0.05) without evident heterogeneity, while this risk was reduced towards the unity in retrospective studies.

When the analysis was restricted to the large study (≥ 500 subjects), pooled risk estimates were statistically significant under allelic (OR = 1.12; *P* = 0.004), homozygous genotypic (OR = 1.25; *P* = 0.005) and dominant (OR = 1.25; *P* = 0.001) models with borderline heterogeneity, while an opposite yet nonsignificant association was identified in the small studies (< 500 subjects).

### CETP C-629A polymorphism and lipid changes

Table 3 presents the overall analyses of *CETP* C-629A polymorphism with circulating lipid changes under both homozygous genotypic and dominant models. An increase in circulating CETP was observed for carriers of the -629CC genotype (WMD: 0.70 μg/mL; 95% CI: 0.30 to 1.10; *P* = 0.001) or -629C allele (WMD: 0.45 μg/mL; 95% CI: 0.25 to 0.65; *P* < 0.001) relative to the -629AA homozygotes, with evident heterogeneity. By contrast, there was a reduced yet nonsignificant trend in circulating triglycerides for -629CC genotype or -629C allele carriers, and the probabilities of heterogeneity and publication bias were low.

**Table 3:** Overall analyses of *CETP* gene C-629A with circulating lipids under both genotypic and dominant models

Lipids	Genetic models	Studies	WMD	95% CI	*P*	*I*^2^ (%)
CETP	Genotypic	4	**0.70**	**0.30 to 1.10**	**0.001**	83.1
	Dominant	4	**0.45**	**0.25 to 0.65**	**< 0.001**	69.3
Triglycerides	Genotypic	9	−2.11	−13.30 to 9.07	0.711	12.9
	Dominant	9	−0.77	−12.41 to 10.86	0.896	42.3
HDL-C	Genotypic	16	**−4.36**	**−7.20 to −1.51**	**0.003**	84.2
	Dominant	16	**−3.65**	**−5.59 to −1.70**	**< 0.001**	78.7
LDL-C	Genotypic	9	9.60	−0.60 to 19.80	0.065	74.8
	Dominant	9	7.03	−0.62 to 14.67	0.072	69.6
Apo-AI	Genotypic	3	−0.75	−6.60 to 5.10	0.800	0.0
	Dominant	3	−3.66	−7.76 to 0.43	0.079	0.0
Apo-B	Genotypic	3	4.77	−3.34 to 12.87	0.249	0.0
	Dominant	3	4.91	−1.10 to 10.91	0.109	0.0

Notes: WMD, weighted mean difference; 95% CI, 95% confidence interval.

Circulating HDL-C was significantly reduced in carriers of the -629CC genotype (WMD: -4.36 mg/dL; 95% CI: -7.20 to -1.51; *P* = 0.003) or -629C allele (WMD: −3.65 mg/dL; 95% CI: -5.59 to -1.70; *P* < 0.001) when compared with the -629AA homozygotes, with moderate heterogeneity and a low probability of publication bias. The -629CC genotype or -629C allele was associated with higher circulating LDL-C than the -629AA genotype, while no significance was reached. Similarly, the -629CC genotype or -629C allele was associated with lower Apo-AI but higher Apo-B than the -629AA genotype, with no observable heterogeneity.

### Meta-regression analyses

To further seek possible causes of clinical heterogeneity, meta-regression analyses that modeled age, male gender, BMI, smoking, dyslipidemia, hypertension, diabetes, circulating triglycerides, total cholesterol, HDL-C, LDL-C, CETP, Apo-AI and Apo-B if available under study were conducted, and none of these factors contributed significantly to the association of *CETP* C-629A polymorphism with CHD risk (all *P* > 0.05).

### Cumulative and influential analyses

Cumulative analyses by ascending publication years indicated no substantive change in the direction of effect estimates with the addition of subsequent studies (Supplementary Figure S1). In addition, influential analyses confirmed the stability of overall effect estimates (Supplementary Figure S2).

## DISCUSSION

The objective of this meta-analysis was to evaluate the association of *CETP* C-629A polymorphism with the risk of CHD and lipid changes by summarizing data from 17 articles. The key finding of this study was that the -629C allele was significantly associated with an increased risk of CHD in Caucasians, and this association may be mediated by its phenotypic regulation on circulating CETP and HDL-C. The importance of the current study lies in deepening our understanding of the functional aspects of *CETP* genetic variation involved in the pathogenesis of CHD.

Lately, a large comprehensive meta-analysis choosing *CETP* gene TaqIB (rs708272) polymorphism as an instrument has demonstrated that circulating CETP may play a causal role in the pathophysiology of CHD [25], although there are still some unresolved issues revolving around the prerequisites of Mendelian randomization analysis [26], such as pleiotropic impact of genetic polymorphism under study and linkage disequilibrium with another locus that differently modifies circulating CETP. Nevertheless, it still remains an open question to interrogate *CETP* genetic loci associated with CHD risk and responsible for the changes of biologically relevant lipids. The conduct of this meta-analysis therefore represents a supplement to medical research and deepens our understanding of the genetics of CHD.

As indicated in this meta-analysis, *CETP* C-629A mutation can alter susceptibility to CHD in Caucasian populations, at least in part, through its phenotypic regulation on circulating CETP and HDL-C. Several cautionary notes regarding the interpretation and extrapolation of this finding should be sounded. First, genetic heterogeneity across ethnicities is a common phenomenon gripping a majority of association studies. It is of interest to found that *CETP* -629C allele was significantly associated with an increased risk of CHD only in Caucasians, while this association was reversed to be protective in Asian and Middle Eastern populations. As a matter of fact, linkage disequilibrium patterns are generally believed to be diverse across races or ethnicities. For example, the linkage of a genetic variant with another functional variant was usually strong in one ethnic group but weak or nonexistent in another [27]. Second, experimental data suggested the close association of *CETP* genetic alterations with increased large cholesterol-enriched HDL particles [28]. Moreover, the fact that simple measurement of circulating HDL-C may not always reflect the potential cardioprotective activity of HDL particle, which might be dysfunctional in spite of high HDL-C can by no means be ignored [29]. It is widely recognized that cholesterol-overloaded HDL particle can not only decrease the hepatic selective uptake of cholesterol from HDL particle, but also exert a defective effect on efflux potential of cholesterol from extra-hepatic cells [30–32]. In addition, some pharmacological agents such as CETP inhibitors [33] and Niacin [34] that raise circulating HDL-C simultaneously increased the levels of cholesterol-overloaded particles. Third, the association of *CETP* C-629A polymorphism with CHD risk had a biological basis, as this polymorphism also accounted for the changes of circulating CETP concentrations. In addition, this association was strengthened after restricting analysis to the prospective and large studies, which further verifies the robustness of our meta-analytic findings.

A number of possible limitations should be recognized for this meta-analysis. First, only summary data from published papers were abstracted, and it could yield further insights if individual participant data were analyzed. Second, we were unable to glean various potential confounders (smoking, dyslipidemia, hypertension and diabetes) from all eligible studies, and only five studies provided complete confounding data. We adopted meta-regression analyses in an attempt to account for this limitation, while no significance was identified. Third, only one promoter polymorphism, C-629A in *CETP* was meta-analyzed, which is clearly not sufficient to support the contributory role of *CETP* in the pathogenesis of CHD and lipid regulation, as other polymorphisms in or flanking CETP might synergize or antagonize the impact of C-629A. Fourth, the association of *CETP* C-629A polymorphism with CHD risk and circulating lipid changes is not based on the same dataset due to limited number of qualified studies. Fifth, as with most meta-analyses, publication bias might be possible because only published articles were retrieved and the ‘grey’ literature (articles in languages other than English) was not reviewed. In view of these limitations, the jury must refrain from jumping at a conclusion until further verification of our findings in large, long-term, well-designed prospective studies.

Taken together, our meta-analytic findings demonstrate that the -629C allele was significantly associated with an increased risk of CHD in Caucasians, and this association may be mediated by its phenotypic regulation on circulating CETP and HDL-C. Although further investigations are required to elucidate the molecular mechanisms of *CETP* C-629A polymorphism underlying CHD, future studies on the relationship between *CETP* genetic defects and CHD susceptibility need to focus on gene-to-environment interactions, especially on the impact of the -629C allele on circulating lipid changes.

## MATERIALS AND METHODS

This meta-analysis of observational studies was conducted according to the PRISMA (Preferred Reporting Items for Systematic Reviews and Meta-analyses) statement [35]. All observational studies included were reported to obtain ethical approvals from the Ethics Committees of local institutions or departments.

### Search strategy

PubMed/Medline, Embase, Scopus and Google Scholar electronic resources were searched on July 21, 2016 to seek articles of potential relevance, by using subject headings (‘cholesteryl ester transfer protein’ or ‘cholesterol ester transfer protein’ or ‘CETP’ and ‘coronary heart disease’ or ‘isch[a]emic heart disease’ or ‘myocardial infarction’ or ‘atherosclerosis’ or ‘arteriosclerosis’ or ‘coronary artery disease’ or ‘coronary disease’) and (‘polymorphism’ or ‘variant’ or ‘variation’ or ‘mutation’ or ‘SNP’). The bibliographies of retrieved articles were also reviewed for articles that might be missed.

### Eligibility assessment

The eligibility of each article was justified by two investigators (Shouwei Lin and Rong Lin) by reading the title and abstract, and if necessary the full text. Meanwhile, the period and location, if available, for study subjects collected were recorded to judge whether there were multiple publications from the same study. If so, the publication with a larger sample size was retained.

To be more specific, three inclusion criteria were proposed: (1) only English-language publications were considered; (2) the association of *CETP* C-629A polymorphism with CHD risk or circulating lipid changes was evaluated; (3) the absolute counts of C-629A genotypes between CHD patients and controls or the circulating lipid concentrations across C-629A genotypes were provided. In addition, articles were not taken into account if they were conference abstracts/posters, case reports, editorials and narrative/systematic reviews.

### Data extraction

The same two investigators (Shouwei Lin and Rong Lin) independently extracted data from each qualified article according to a jointly formulated protocol, including first author's surname, publication year, ethnicity, diagnostic criteria of CHD including coronary stenosis or myocardial infarction, study design (retrospective or prospective design), source of controls (hospitals or populations), matched situation, sample size, absolute genotype counts of *CETP* C-629A polymorphism between CHD patients and controls or mean (standard deviation) concentrations of circulating CETP, triglycerides, HDL-C, LDL-C, Apo-AI and Apo-B across C-629A genotypes, as well as age, male gender, body mass index (BMI), smoking, dyslipidemia, hypertension, diabetes, circulating triglycerides, total cholesterol, HDL-C, LDL-C, CETP, Apo-AI and Apo-B, if available, between CHD patients and controls. For the sake of consistency, circulating triglycerides, total cholesterol, HDL-C, LDL-C, Apo-AI and Apo-B were expressed in mg/dL and CETP in μg/mL.

### Statistical analysis

Unadjusted odds ratio (OR) and weighted mean difference (WMD), along with 95% confidence interval (95% CI) were calculated by using a random-effects model with the DerSimonian & Laird method to pool individual effect-size estimates under all circumstances. The magnitude of statistical heterogeneity across studies was represented by the inconsistency index (*I*^2^) statistic (range: 0% to 100%). Statistical heterogeneity was reported to be significant if the *I*^2^ statistic is over 50%, which is a generally accepted cutoff value [36].

To explore the possible causes of clinical heterogeneity, grouping all qualified studies by gender, ethnicity, CHD subtype, control source, study design, matched status and sample size was conducted, separately. In addition, clinical heterogeneity was explored by meta-regression analyses that incorporated all available discrete and continuous variables under study.

To see how effect estimates have shifted over time, cumulative analyses were performed in time sequence with each sub-analysis incorporating one additional study. To examine the robustness of overall estimates, influential analyses were undertaken by excluding each study from the analysis to seek its impact on the overall findings.

The assessment of publication bias was made by the Begg's funnel plots and Egger's asymmetry tests. The Egger's test can inspect funnel plot asymmetry by determining whether the intercept deviates significantly from zero when regressing the standardized effect estimates against their precision [37]. *P* < 0.10 was chosen for the significance of Egger's tests. Data were statistically analyzed by the STATA software version 14.0 for Windows 10.0 (StataCorp, College Station, TX, USA).

## SUPPLEMENTARY MATERIALS FIGURES AND TABLES




