# Supplementary material for: A meta-analytic evaluation of cholesteryl ester transfer protein (CETP) C-629A polymorphism in association with coronary heart disease risk and lipid changes
Source: Oncotarget. 2016 Oct 25;8(2):2153–63. doi: 10.18632/oncotarget.12898 (PMC5356788; doi:10.18632/oncotarget.12898)
Supplement: Supplementary file 1 [file oncotarget-08-2153-s001.pdf]

A meta-analytic evaluation of cholesteryl ester transfer protein (*CETP*) C-629A polymorphism in association with coronary heart disease risk and lipid changes

Supplementary Materials

Supplementary Table S1: The baseline characteristics and circulating lipid profiles of all studies for the genotype-phenotype relationship. See Supplementary_Table_S1

Allelic model

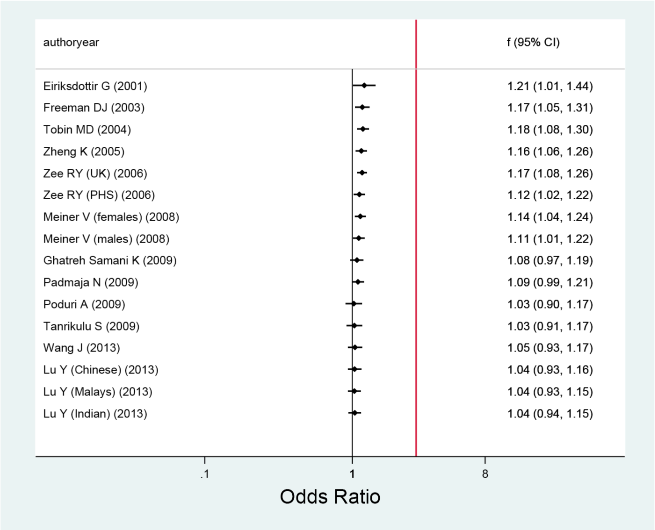

Homozygous genotypic model

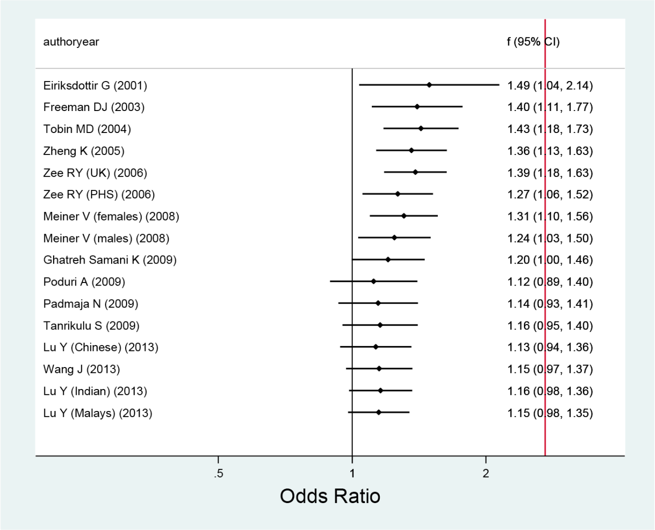

Dominant model

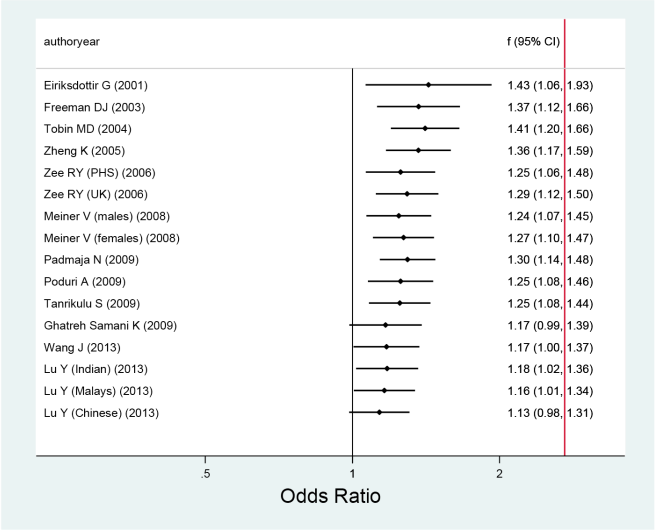

Supplementary Figure S1: Cumulative analyses of *CETP* C-629A polymorphism in susceptibility to CHD under three genetic models.

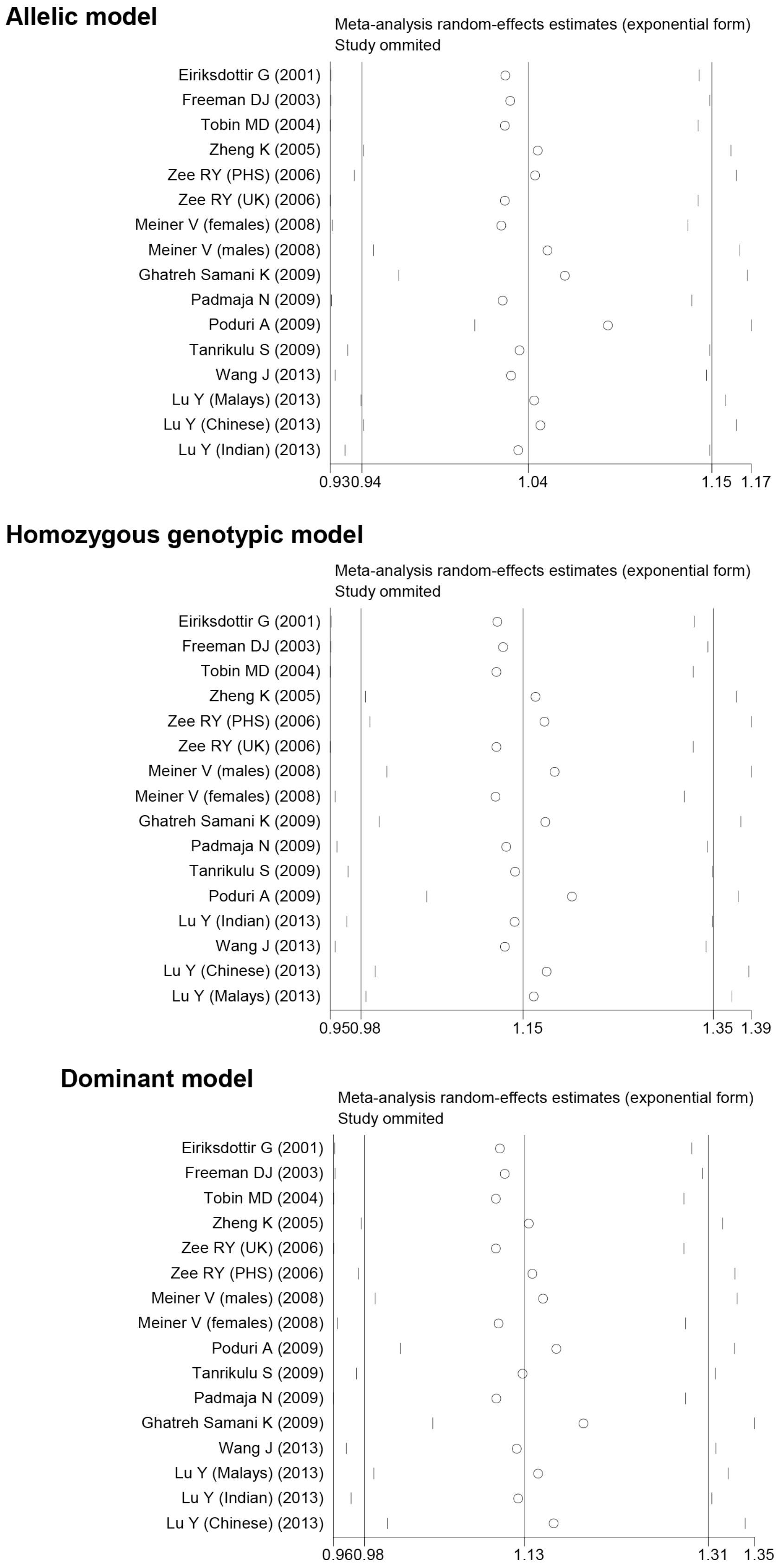

Supplementary Figure S2: Sensitivity analyses of *CETP* C-629A polymorphism in susceptibility to CHD under three genetic models.
